# Manganese Oxidation during Vegetation Burning

**DOI:** 10.1021/acs.est.6c05048

**Published:** 2026-06-01

**Authors:** Shyrill Mae F. Mariano, Lingqun Zeng, Rixiang Huang, Carmen Sánchez-García, Cristina Santin, Jonay Neris, Peng Yang, Lu Ma, Andrew Kiss

**Affiliations:** 1 Department of Environmental and Sustainable Engineering, 1084 University at Albany, 1400 Washington Ave, Albany, New York 12222, United States; 2 European Commission, Joint Research Centre (JRC), Ispra, Varese 21027, Italy; 3 Centre for Wildfire Research, Swansea University, Swansea SA2 8PP, U.K.; 4 Research Institute of Biodiversity (IMIB; CSIC-UnOvi-PA), University Campus of Mieres, Mieres 33600, Spain; 5 Universidad de La Laguna, Tenerife 38206, Spain; 6 National Synchrotron Light Source II, 8099 Brookhaven National Laboratory, Upton, New York 11973, United States

**Keywords:** wildland fires, ash, manganese cycling, speciation, X-ray absorption spectroscopy

## Abstract

Redox recycling of manganese (Mn) plays a key role in organic matter decomposition and nutrient cycling in terrestrial vegetated ecosystems, and it is expected to be changed by fires. This study revealed how Mn is oxidized during vegetation burning, by characterizing the chemical speciation of Mn in fire ash from wildland fires and laboratory burning and evaluating the factors governing its average oxidation state (AOS) and speciation. Manganese in wildland fire ash from different ecosystems showed variable AOS that ranges from 2.5 to 3.3. Laboratory burning experiments showed that Mn oxidation was primarily controlled by fire thermal intensity (temperature × duration) and burning completeness. As heating time increased from 5 min to 5 h at 550 and 700 °C, Mn AOS in the lab-burned vegetation ash increased from 2.7 to 4.0 and the oxidation rate was faster at higher temperature. Diverse Mn species can present in wildland fire ash and differ structurally from biogenic Mn oxides. The oxidized Mn species enable fire ash to mediate oxidative degradation of catechol, demonstrating its potential in mediating organic matter decomposition. This study revealed a new paradigm of Mn redox recycling, as compared to the microbe-mediated Mn redox cycling in the absence of fires.

## Graphical abstract

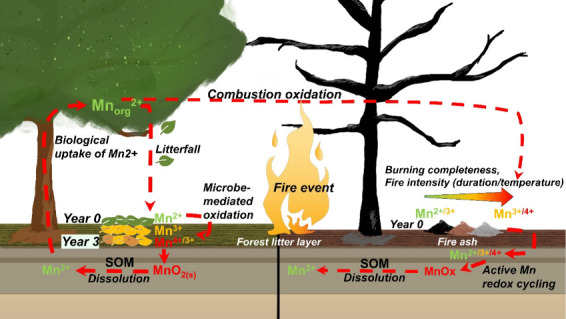

## 1 Introduction

Manganese (Mn) plays an important role in critical ecosystem processes such as plant growth, nutrient cycling, and organic matter (OM) decomposition and stabilization.^1,2^ Specifically, Mn contributes to the enzymatic oxidative degradation of OM through the functional roles of Mn peroxidase,^3^ and Mn minerals mediate abiotic oxidative transformation of OM and OM stabilization through mineral–OM association.^1^ The roles of Mn in these ecosystem processes depend greatly on its redox cycling, which affects its availability for enzymes and its oxidative or adsorptive reactivities toward OM.^4–6^ The chemical forms of Mn experience dynamic changes during its biogeochemical cycling within the soil–plant system, affecting its reactivity and mobility (Figure 1).^2^ First, free or carboxylate-bound Mn­(II) is taken up by plants from pore water and is stored in the plant biomass.^7^ After litterfall, microbes mediate the decomposition of OM (initiated with enzymatic depolymerization),^8^ during which the Mn­(II) in litter is released and gradually oxidized by fungi and bacteria to form Mn­(III)–ligand complexes and insoluble phyllomanganates.^9,10^ Some microbial species can oxidize Mn­(II) to birnessite, a reactive form that can catalyze the oxidation of soluble Mn­(II) into various Mn­(III/IV) oxides.^11^ Soil Mn­(III/IV) oxides mediate oxidative transformation of OM, which results in the reductive dissolution of Mn in the soil and the reduced Mn­(II) becomes soluble and bioavailable.^12,13^ Microbial oxidation of Mn is generally kinetically faster than abiotic mechanisms^14^ but can still be constrained by Mn availability.^2^ Studies in controlled forest environments revealed that the complete oxidation of Mn­(II) to Mn­(IV) may take up to six years.^2^

**Figure 1:**
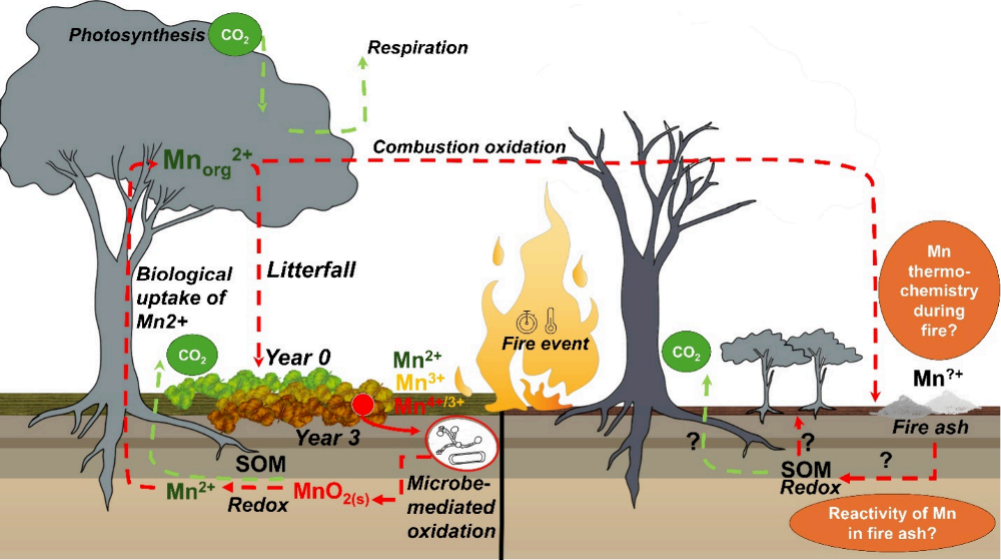
Schematic illustration of the coupled Mn (red arrow) and C (green arrow) cycling in the plant–soil continuum in the absence and presence of fire. The goal of this study is to determine how Mn is thermochemically transformed during wildland fires.

Fire is a common ecological disturbance in most terrestrial ecosystems that alters many biogeochemical processes including carbon (C) and nutrient cycling.^15–17^ Through the burning of biomass, fire concentrates a large fraction of aboveground nutrient pools in the ash,^18^ influencing the mobility and bioavailability of nutrients.^19^ Herein, fire ash is referred to as the particulate residue deposited on the ground after fire and is generally a mixture of inorganic and charred organic materials.^19^ Previous studies on ash focused on the thermochemical transformation of macronutrients such as nitrogen and phosphorus,^20,21^ and their postfire cycling in soils.^17,22,23^ Recent studies started to look into Mn and revealed the presence of Mn oxides in fire ashes and soils in burned environments.^24,25^ However, to our knowledge, no study has characterized the thermochemistry of Mn during wildland fires and its ecological impacts, despite the important roles of Mn in critical ecosystem processes.^6,26^ In particular, fires occur across diverse ecosystems with varying vegetation types and fire severities, yet their effects on Mn cycling remain poorly understood (Figure 1).

In this study, we aim to determine the thermochemical transformation of Mn during vegetation burning and the main factors controlling its speciation in fire ashes. We hypothesize that (1) Mn will be oxidized during vegetation burning and present at variable oxidation states in fire ash and (2) Mn oxidation during vegetation burning is primarily controlled by fire thermal conditions, specifically burning temperature and duration. We test these hypotheses by characterizing ash samples generated from wildland fires and laboratory heating experiments. To explore the separate effects of fire temperature and duration (thermodynamic and kinetic controls), we resort to laboratory heating of pure Mn compounds and a uniform biomass of different sources.

## 2 Materials and Methods

### 2.1 Sampling of Wildland Fire Ash and Biomass

Nine wildland fire ash samples collected from diverse ecosystems (boreal forest, temperate conifer forest, temperate eucalypt, heathland, savanna, and pine barrens) were used to study the variations of Mn oxidation state among wildland fire ash. The samples were labeled by the ecosystem type or location. Details on the collection and composition of these samples can be found in the Supporting Information (Table S1 and Text S1) and our previous work.^19,27^ Our previous result demonstrated the variability of Ca and P speciation in these ash samples, as a result of interacting fuel biomass composition (based on Ca/P ratio) and fire thermal conditions.^27^ Ash samples from prescribed fires at the Albany Pine Bush Preserve (APBP, Albany, New York) were collected from sites dominated by woody vegetation and grass, respectively. Two of them were fractionated with a 125 μm pore size sieve into coarse and fine fractions, which differed in burning completeness (measured by loss on ignition, LOI). Loss on ignition (LOI) of samples from size fractionation was measured by burning the sample at 550 °C for 5 h in air, and the percentage of mass loss was reported as the LOI.

Coniferous tree biomass used for the laboratory heating experiment (below) included (1) white pine needle (*Pinus strobus*) and (2) cone, leaf, and stem of black spruce (*Picea mariana*). The biomass consisted of fresh litter that was pooled from randomly distributed sampling spots (*n* = 3) at the APBP. The biomass types were selected because (1) white pine and black spruce are widely distributed and representative coniferous trees in the temperate and boreal forests, respectively, in North America^28,29^ and (2) although the focus is to evaluate the effects of thermal conditions, the selected sample set also enables the comparison among biomass types.

### 2.2 Laboratory Heating Experiments

The effects of burning conditions (temperature and duration) and biomass sources on Mn oxidation and speciation were studied using (1) temperatures 550 and 700 °C, (2) durations 5, 20, 30, and 300 min, and (3) biomass white pine needle (WPN), black spruce needle (BSN), black spruce stem (BSS), and black spruce cone (BSC). The selected temperature and duration are within the common range observed for wildland fires and primarily regulate the burning completeness of ash.^30–32^ The relatively long duration of 300 min was chosen to represent an extreme state where the biomass is completely burned (with no further mass change), and thermochemical reactions have reached equilibrium. The selected biomass covers different plant species and compartments.

Dry biomass was shredded with a blender before burning. The ground biomass was placed into a preheated furnace at the designated temperature and durations and was quickly removed as the targeted durations were reached. Replicate experiments (*n* = 3) were performed under each burning condition. The mass of the preburned biomass and postburned ash was recorded, and the mass recovery (%) was determined.

Two additional experimental sets comprised of pure Mn­(II)-acetate and WPN were combusted at 350, 450, and 600 °C for 5 h in air to further investigate the difference in oxidation pathways between a pure organic Mn compound and biomass Mn. In this burning experiment, 5 to 15 g samples were weighed in either glass vials or aluminum cups and placed in a preheated furnace for combustion at the designated duration.

### 2.3 Chemical Composition Measurement

Total Mn ([Mn]_total_) of the biomass and fire ash was determined by digestion in aqua regia and then analyzed by inductively coupled plasma optical emission spectroscopy (ICP-OES, Agilent 5800). Bioavailable Mn ([Mn]_Pyro_%) in the fire ash was determined by a modified pyrophosphate extraction method, because pyrophosphate primarily extracts both Mn^2+^ and Mn^3+^ in the forms of soluble and organic-complexed Mn,^2^ while it is unable to extract silicate-bound and crystalline Mn oxides.^33,34^ The extracted Mn was quantified by an ICP-OES analysis. Details of the analysis can be found in Text S2.

#### Mn Oxidation State Determined by the Leucoberbelin Blue (LBB) Colorimetry Method

The Mn oxidation state (Mn_LBB_AOS) of the ash samples was initially evaluated through a modified rapid colorimetric determination using a 0.04% Leucoberbelin blue (LBB) solution.^35^ Ash samples ranging between 20 and 50 mg were mixed with 0.4 mL of LBB solution and were allowed to react for 20 min in the dark. The reacted solutions were diluted to 2.0 mL, filtered, and analyzed for Mn using a UV–vis spectrophotometer (UV-1900, Shimadzu, Japan) at 620 nm.

### 2.4 Oxidative Reactivity Measurement

The redox activity of Mn in ash was evaluated by a batch reaction with catechol. Catechol represents model phenolic compounds and is commonly used to study the reactivity of Mn oxides.^36,37^ The rate of catechol oxidation was studied by mixing 40 mL of 1 mM catechol solution in deionized water with WPN ash (from 5 h heating). A WPN ash suspension with deionized water served as control. The solution was placed on a horizontal shaker (170 rpm) for 24 h, and a 1 mL aliquot was sampled at different time points (0, 1, 3, 5, 20, and 24 h). WPN ash filtered at different time points was frozen immediately and freeze-dried and saved for spectroscopic analysis. An additional catechol reaction experiment was conducted with prescribed fire and laboratory burning ashes under higher concentration (10 mM) and longer duration (14 days and 21 days, respectively). After the reaction, the ash was rinsed with ultrapure water and dried. The reacted ashes were finely ground for the spectroscopic analysis.

The effect of Mn content in ash on catechol oxidation was also investigated by mixing three different loads (10, 20, and 30 mg) of WPN ash ([Mn] = 25 g/kg) and APBP-W1 ash ([Mn] = 5 g/kg) with 2 mL of 1 mM catechol, and the experiment lasted for 18 h. For both experiments, the solutions were filtered and diluted to appropriate concentrations before scanning by a UV–vis spectrophotometer from 200 to 900 nm to detect change in the catechol peak (275 nm) and formation of other dissolved organic intermediates and products confirmed by a change in spectral shape.^38^

### 2.5 Synchrotron X-ray Spectroscopic and Spectro-Microscopic Analyses

#### Bulk X-ray Absorption Analysis

Manganese K-edge X-ray absorption spectroscopy (XAS) data were collected at beamline 11-2 at the Stanford Synchrotron Radiation Lightsource (SSRL) and at beamlines 6-BM and 7-BM at the National Synchrotron Light Source II (NSLS-II). The XAS spectra were collected from the energy range of 6310.0 to 6937.2 eV at SSRL and 6339.0 to 7084.0 eV at NSLS-II. Extended X-ray absorption fine structure (EXAFS) was collected for selected samples with relatively high Mn contents using 0.05 k for energy beyond 6564.0 eV. Multiple scans were collected for each sample. The average oxidation states (AOSs) of the samples were determined through the Combo method using the first derivative, and spectra of a range of heterovalent Mn species were used (Figure S1 and Table S2).^39^ EXAFS data analysis was done in ARTEMIS software^40^ for suitable samples to identify specific Mn species. Further details on data collection at the beamlines and data analysis are fully described in Text S3.

#### Micro X-ray Fluorescence (μ-XRF) Imaging and μ-XAS Analysis

Two prescribed fire ash samples (wood and grass) were selected for μ-XRF imaging and μ-XAS analysis at NSLS-II Beamline 5-ID.^41^ The fire ash was gently pressed on Kapton tape. Sample-loaded tapes were mounted on a sample stage, and the tape-covered side was raster-scanned under the beam at an energy of 10 keV and to acquire XRF maps of 200 μm × 200 μm with a pixel size of 1 μm by 1 μm. Processing of the image data was done using the SMAK 3.0 package (Sam’s Microprobe Analysis Toolkit).^42^ At selected spots, Mn K-edge μ-XANES spectra were collected and fitted similarly by the Combo method, to determine the Mn AOS.

## 3 Results

### 3.1 General Chemistry of Wildland Fire Ash

Key physicochemical properties such as elemental composition and stoichiometry, reflecting the variations in biomass sources and fire conditions across ecosystems, were reported in our previous work.^43^ Overall, ash samples from ecosystems dominated by woody plants (e.g., boreal forest, temperate conifer, or eucalypt forests) exhibited relatively high Ca/P ratios, while those from heathland and savanna possess relatively low Ca/P ratios.^44^ The total C content ranges from 7 to 53 wt %, indicating a variation in burning completeness.

Mn content among the ash samples varied across different ecosystems and wildland fire conditions (Table S1). Prescribed fire ash from pine barrens contained the highest concentrations of Mn (4500–5400 mg/kg), which were a magnitude higher than the Mn concentrations in wildfire ash from the savanna, boreal forest, temperate eucalypt, and temperate moorland ecosystems (300–500 mg/kg). The portion of bioavailable Mn (Mn_pyro_, % of total Mn) was the highest in boreal and eucalypt ash samples (55–62%), followed by Heath-UK and APBP Grass ash samples (39–41%), and then Heath-Sp, Savanna, and APBP W2 ash samples (30–33%), and lastly temperate conifer and APBP-W1 ash samples (17–19%). Variation in pyrophosphate-extractable Mn reflects a potential difference in Mn speciation in the fire ash, which is analyzed below.

### 3.2 Manganese Speciation in Wildland Fire Ash

All samples showed varying and relatively high oxidation states (Mn_XAS_ AOS = 2.5 to 3.3, Figure 2), compared to the Mn AOS of living biomass and fresh litter (Mn AOS = 2.1 to 2.2).^2,9^ The LBB colorimetry method similarly showed high oxidation states, with Mn_LBB_ AOS ranging from 2.4 to 3.5 (Table S1). Moreover, combo fitting suggests that wildland fire ash simultaneously consists of multiple Mn species of different oxidation states (Table S3). Mn^3+^ species were present in all of the ash samples, with abundance ranging from 20 to 64%. The abundance of Mn^4+^ species was 18 to 67% in most samples, whereas the abundance of organic Mn (as represented by Mn^2+^ species) ranged from 9 to 53%. Overall, the relative abundance of Mn^2+^ or the sum of Mn^2+^ and Mn^3+^ in fire ash samples correlated linearly with the % of Mn_pyro_ (*R* ^2^ = 0.654 and 0.522, respectively). This suggests that low-valence Mn species contribute to the pyrophosphate-extracted Mn.

**Figure 2:**
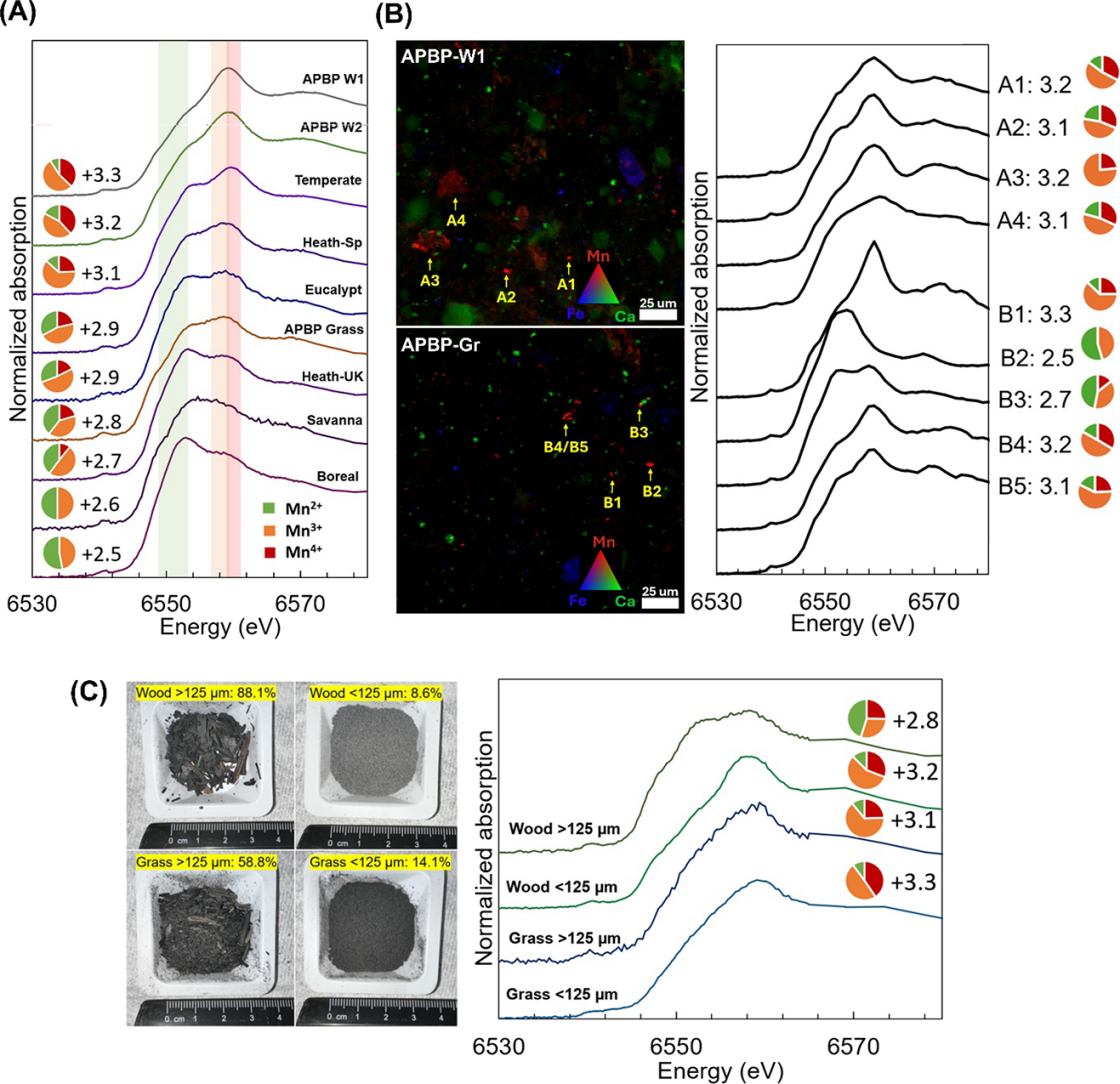
(A) Mn K-edge XANES spectra of wildland fire ash samples and their Mn AOS. Absorption edge energy range for Mn^2+^_,_ Mn^3+^, and Mn^4+^ references are highlighted. (B) Tricolor μ-XRF maps of two APBP prescribed fire ashes and corresponding Mn XANES and AOS of highlighted Mn particles. (C) Images of the two size fractions of APBP prescribed fire ashes (dominated by woody plants and grass, respectively) and the corresponding Mn K-edge XAS and AOS. Included in the highlighted labels are the LOI (%) for the size fraction.

We performed spectro-microscopic analysis on two selected ash samples (APBP W2 and Grass), which showed more detailed Mn speciation (Figure 2B). Micro-XRF images showed that the ashes contained discrete fine Mn particles (with size <∼3 μm) and relatively large particles with colocalized Ca and Fe. The fine particles are most likely particles with a high degree of burning completeness composed primarily of inorganic materials and are therefore relatively homogeneous in composition. The large particles are primarily incompletely burned particles, evidenced in their relatively low intensity (diluted by organics). Similar to the bulk speciation, the Mn particulates are also chemically heterogeneous, varying in Mn AOS and consisting simultaneously of multiple valent Mn. For example, Mn AOS of particles in APBP W2 ranged between 3.09 and 3.23, and in APBP Grass ranged between 2.45 and 3.29.

Size fractionation of the two ash samples substantiated that the relatively fine fraction (<125 μm) has a higher degree of burning completeness (more inorganics) and higher Mn AOS values, compared to those of the coarse fraction (Figure 2C). Specifically, the fine fraction has relatively low LOI values (8.6 and 14%) than the less combusted coarse fraction (88.1 and 58.8%), regardless of biomass source (grass or woody biomass). Similarly, the fine fraction has high Mn AOS values of 3.2 and 3.3, compared to 2.8 and 3.2 for the coarse fractions. Combo fitting of the Mn XANES data showed a higher percentage of Mn^3+^ and Mn^4+^ species in the fine and high combusted fraction compared to more Mn^2+^ in the less combusted fraction (Table. S3).

### 3.3 Manganese Chemistry in Ash from Laboratory Heating Experiments

Burning at variable temperatures and durations resulted in ash with varying degrees of burning completeness, measured by mass recovery (%). Overall, mass recovery decreased as heating duration or temperature (or duration × temperature) increased (Figure 3A,B). For example, mass recovery of WPN ash at 550 °C decreased from 16.5 to 4.6%, as heating duration increased from 5 to 30 min. At 700 °C, a high degree of burning completeness was reached at even 5 min. It is worth noting that burning behavior (time-dependent mass recovery) can differ among biomass types, depending on factors such as chemical composition and physical forms (evaluation of their effects is out of the scope of this study).

**Figure 3:**
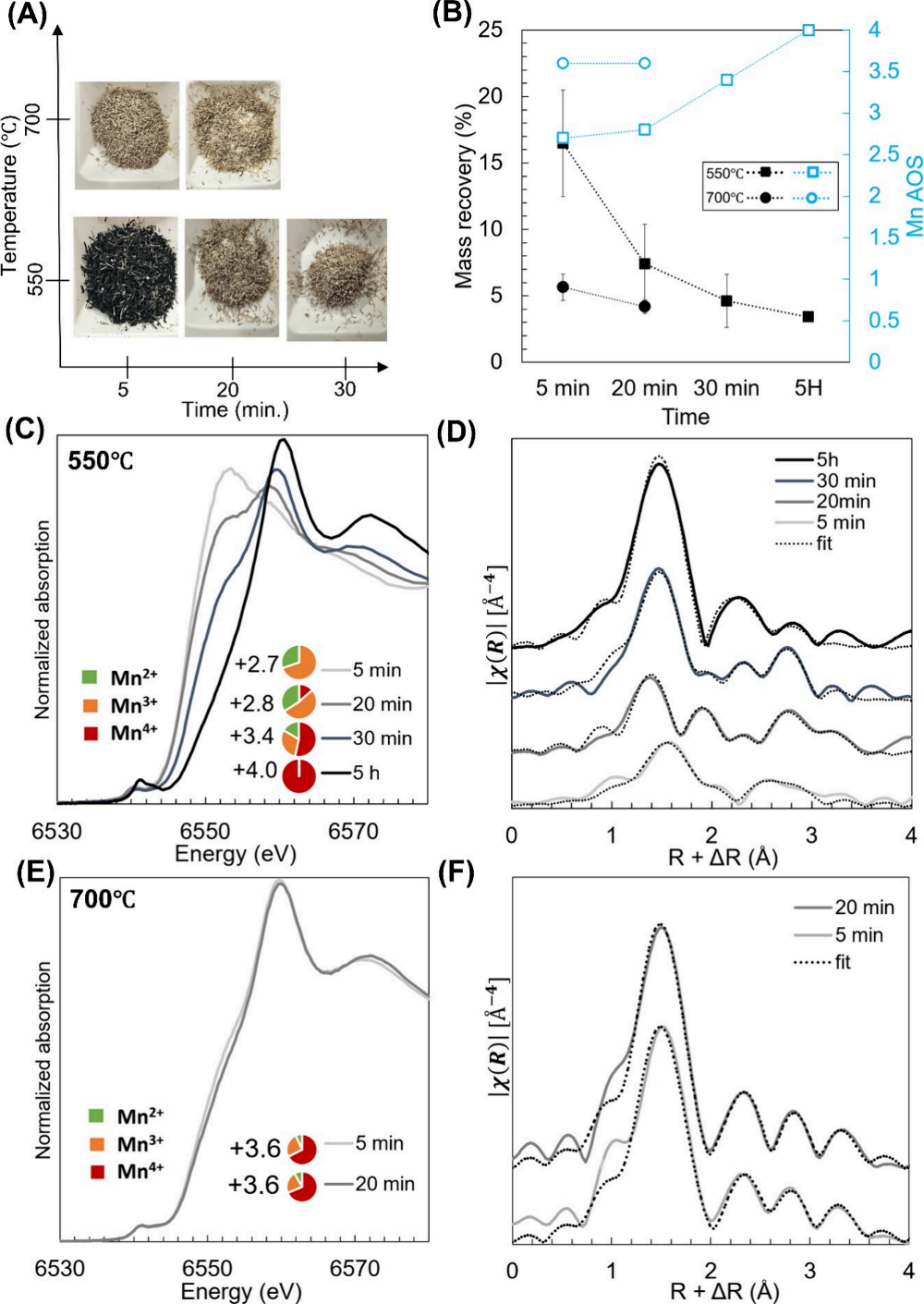
(A) Images of ash from different burning durations (5, 20, and 30 min) and temperatures (550 and 700 °C) and (B) the corresponding mass recovery (%) and Mn AOS. Manganese K-edge XAS of WPN ash from heating at 550 °C (C) and 700 °C (E), and the corresponding EXAFS shell-by-shell fitting for an *R* range of 1 to 4 Å (D and F, respectively).

Manganese AOS of the resulting ash increased as heating duration (or degree of burning completeness) increased, reflecting a kinetic-controlled Mn oxidation. For example, Mn AOS of WPN ash generated at 550 °C increased gradually from 2.7 at 5 min, to 2.8 at 20 min, and then 3.4 at 30 min, until 4.0 at 5 h (Figure 3B). Similarly, Mn AOS of black spruce needle and stem also increased with increasing durations, ultimately reaching 4.0 at 5 h (Figure 4). The rate of Mn oxidation was faster at 700 °C, with Mn AOS of the WPN ash reaching 3.6 at both 5 and 20 min (Figure 3C). We also heated WPN at 450 and 600 °C for 5 h and Mn AOS of the ash all reached 4.0 (Figure S3). The data suggests that Mn^4+^ is the equilibrium state in ash from prolonged heating at temperature from 450 to 700 °C.

**Figure 4:**
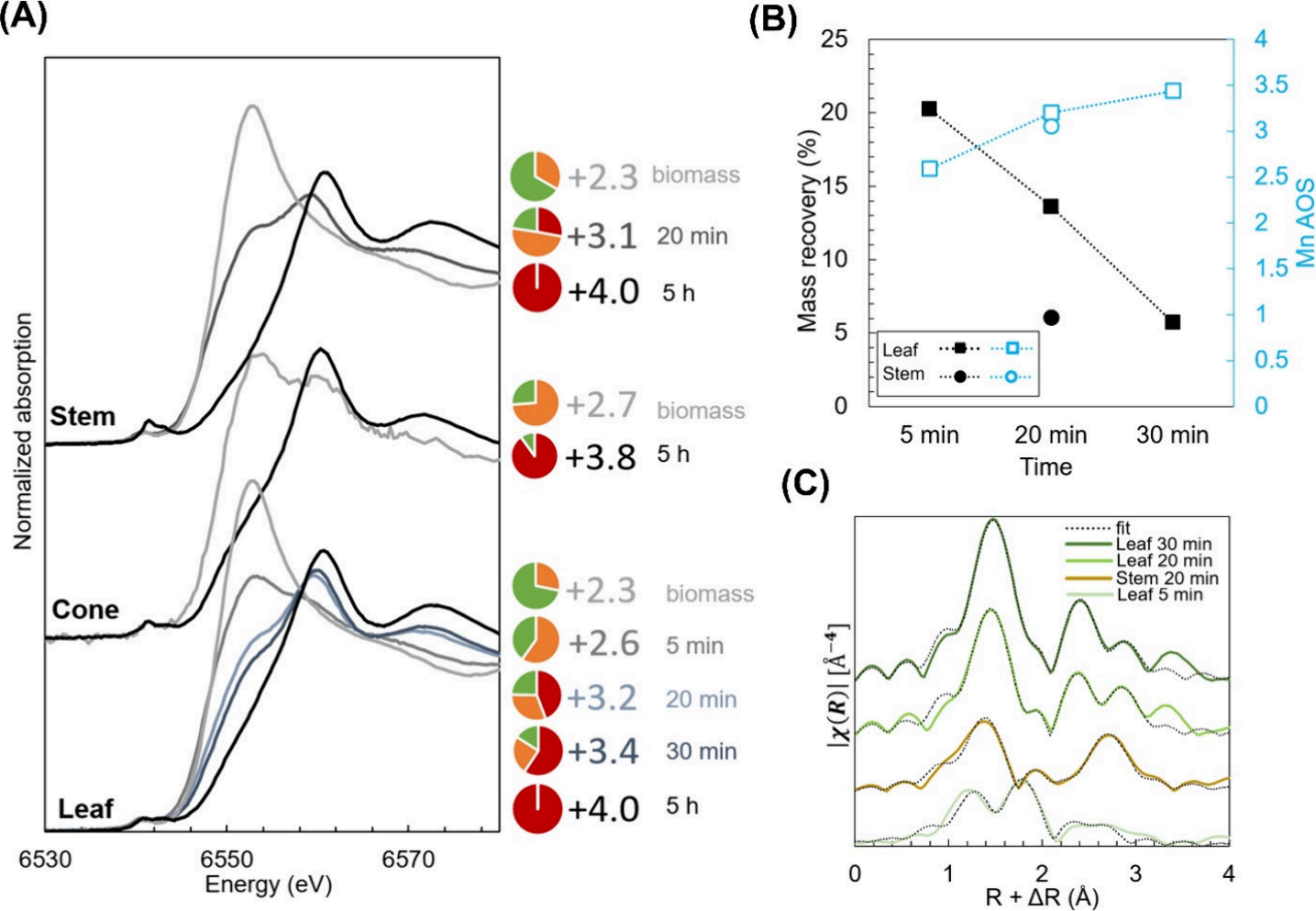
(A) Mn K-edge XAS of ash of black spruce (BS) compartments from heating at 550 °C for varying durations and the corresponding Mn AOS. (B) Mass recovery (%) and Mn AOS vs burning duration for the heating of black spruce compartments. (C) EXAFS shell-by-shell fitting for selected black spruce ash samples.

Results from EXAFS shell-by-shell fitting showed that Mn in ash from low-intensity heating (with AOS <3.4) exhibited four coordinated and two coordinated O atoms between ∼1.9 and ∼2.3 Å, which is distinctive of the Jahn–Teller distortion of Mn­(III) (Figures 3D and 4C). There are also between one and six Mn atoms within ∼2.8 and ∼3.1 Å (Figures 3D and 4C and Table S4). The CN and *R* values are characteristic of the Mn­(III) oxyhydroxide group,^45^ and the large variations in the farther Mn shells reflect increasing disorder for ash from low-intensity burning. In comparison, Mn in ash from high-intensity heating (and AOS >3.4) showed Mn–O_1_, Mn–Mn, Mn–Ca, and Mn–O_2_ bonds, which correspond to the Ca_2_Mn_3_O_8_ structure that was identified for all completely burned ash (5 h heating, AOS = 4.0) (Figures 3F and S3 and Table S4).

### 3.4 Redox Reactivity of Mn in Ash

The oxidative reactivity of Mn in fire ash was demonstrated in a catechol oxidation experiment, in which catechol was degraded in the presence of a lab-burned WPN ash (550 °C and 5 h) and a prescribed fire ash (APBP-W1) (Figure 5). The kinetics experiment showed that the characteristic absorption of catechol (*A*_275nm_) diminished within 1 h in the presence of WPN ash ([Mn] = 25 g/kg and [Fe] = 3.2 g/kg) (Figure 5A). Degradation of catechol was rapid and exhibited apparent first-order kinetics (*k* = 0.3 h^–1^) within the first 5 h. After which, the reaction reached a plateau until the end of the 24 h reaction time, suggesting the formation of nonreactive products or limitation in reactive sites. This behavior is consistent with a previously reported two-step reaction between catechol and metal oxides.^46^ The reactivity of ash Mn was further tested by batch experiments with variable amounts of the ash. As the UV absorption data showed (Figure 5B), decrease in the characteristic absorption of catechol correlated linearly with ash loading, and WPN ash caused more reduction than APBP-W1 ([Mn] = 5 g/kg and [Fe] = 4.3 g/kg). Manganese K-edge XANES spectra of the pristine and reacted ash samples showed that Mn in the ash was gradually reduced, especially after 14 d (Figure 5C). However, no reduction was observed for ash mixed with deionized water. Moreover, other redox-active elements such as Fe and nickel are far less abundant than Mn. Data of both the aqueous and solid phases (UV absorption and Mn XAS data) confirm that the oxidized Mn in ash possesses oxidative reactivity toward organic structures.

**Figure 5:**
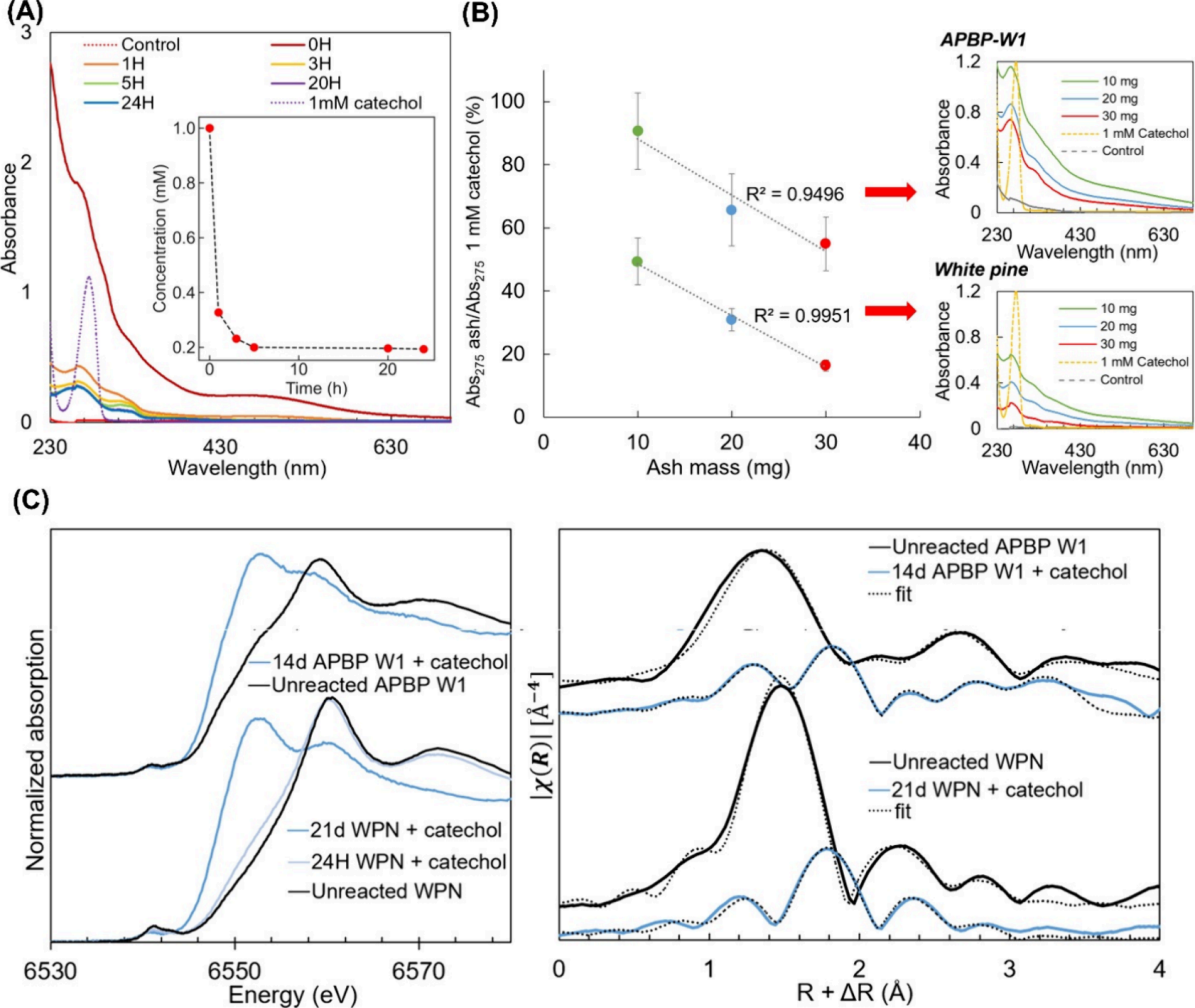
(A) Temporal evolution of UV–vis absorption spectra of 1 mM catechol following reaction with WPN ash from 0 to 24 h. UV–vis absorption of DI water (control) following equilibrium with the ash was small, suggesting that the interference of ash solutes was negligible. Change in catechol concentration over time is shown as an inset, exhibiting the rapid initial degradation of catechol during the first 5 h. (B) Changes of UV absorption of 1 mM catechol in the presence of different amounts of the WPN ash and prescribed fire ash (APBP-W1) after 18 h of reaction, with corresponding UV–vis absorption spectra from 230 to 700 nm. The error bar represents standard deviation (*n* = 3). (C) Mn K-edge XAS spectra of the two ashes following reaction with catechol for variable durations (14 days for APBP-W1, 24 h and 21 days for WPN ash). Corresponding EXAFS fitting for pristine and reacted ashes is shown on the right.

Shell-by-shell fitting of the EXAFS data showed how Mn in fire ash was transformed during catechol oxidation (Figure 5C). Specifically, Ca_2_Mn_3_O_8_ in WPN ash and Mn­(III) oxyhydroxides in APBP-W1 were similarly reduced to a phase close to feitknechtite. Both reacted ashes exhibited the characteristic MnO_6_ octahedral of feitknechtite for the first Mn–O coordination shell, while the outer Mn–O and Mn–Mn shells exhibited a measurable shift in radial distances.

## 4 Discussions

### 4.1 Manganese Thermochemistry during Vegetation Burning

This study systematically characterized the transformation of Mn during vegetation burning and identified the main factors governing Mn speciation in biomass ash. Our results showed that burning temperature and duration primarily regulate Mn oxidation and phase transformation during vegetation burning (Figures 3 and 4), as a result of thermodynamic and kinetic controls.^47^ First, increasing burning durations and temperatures led to increasing Mn AOS and evolving contributions of trivalent and tetravalent Mn species in the ash. Second, complete burning of WPN and black spruce biomass from prolonged heating (5 h) similarly led to the formation of a tetravalent Mn oxide (Ca_2_Mn_3_O_8_). The result suggests that biomass Mn [different Mn­(II) species] is likely transformed into trivalent and/or tetravalent Mn species (e.g., Mn oxyhydroxides such as feitknechtite), and then tetravalent Mn, with Ca_2_Mn_3_O_8_ being the stable species in completely burned ash. Oxidation of the Mn­(II) species in biomass can be activated at temperature down to 450 °C, while kinetics of the reactions increases with temperature.

Heat-induced oxidation of Mn­(II) species and the dependency on temperature and durations have been demonstrated in previous studies on the heating of pure Mn compounds (e.g., inorganic and organic Mn­(II) and various Mn oxides), as demonstrated in the heating of Mn­(II)-acetate (Figure S4).^48–51^ These studies showed that the decomposition and oxidation of different Mn­(II) compounds are activated at different temperatures and may experience different reaction paths. For example, the heating of Mn­(II) glycolate led to the formation of Mn_3_O_4_ at relatively low temperatures (200–450 °C), followed by Mn_5_O_8_, and then α-Mn_2_O_3_ (bixbyite) at temperatures above 500 °C (Figure S5).^49^ In comparison, MnSO_4_ was relatively stable and only oxidized into α-Mn_2_O_3_ at temperatures beyond 625 °C, and then Mn_3_O_4_ at temperatures above 850 °C, and the oxidation was kinetically slow (Figure S5).^48^ Another study showed the formation of Mn_3_O_4_ and MnO_2_ as intermediates, and then α-Mn_2_O_3_ at 550 °C and above, during the heating of organic Mn­(II) and Mn­(II) nitrate.^50^ As the results show, differences in Mn phase evolution existed between vegetation burning and heating of pure Mn compounds (Figures S3 and S4). In particular, α-Mn_2_O_3_ is commonly the stable phase from the calcination of organic and inorganic Mn­(II) above 500 °C in air,^47^ while Ca_2_Mn_3_O_8_ is the determined stable phase for vegetation burning at temperatures between 450 and 700 °C, with feitknechtite being a common intermediate phase. The discrepancy is possibly caused by the chemical heterogeneity of vegetation biomass (compared to pure Mn compounds) and variable and dynamic thermal conditions of vegetation burning that are different from the calcination of pure Mn compounds. First, Mn­(II) in vegetation biomass may possibly exist as diverse organic and inorganic species in a complex organic matrix. Second, biomass burning is a rather dynamic and variable process (even during heating in the furnace), during which the temperature and the matrix change and other thermochemical reactions simultaneously occur. Differences in starting Mn species and thermal conditions will lead to transformation paths (e.g., activation temperature, kinetics, and mineral phases) that are different from the calcination of pure Mn compounds. The matrix property of biomass may be conducive to the formation and stabilization of Ca_2_Mn_3_O_8_ in completely burned plant ash. In living organisms, Mn serves either as an enzyme cofactor or as a metal catalyst in biological clusters.^52^ Mn^2+^ and Ca^2+^ are known to form a CaMn_4_O_5_ cluster that catalyzes the photolysis step of photosynthesis, which requires a large accumulation of Mn^2+^ in the chloroplast alongside Ca^2+^.^53^ In addition, Ca generally is the most abundant metal in plant biomass and form various carbonates in biomass ash.^44^

### 4.2 Heterogeneous and Variable Mn Chemistry in Fire Ash as Related to Wildland Fire Behaviors

Our results showed that Mn speciation in wildland fire ash is highly heterogeneous and variable across ecosystems in terms of overall Mn AOS and speciation (Figure 2). The heterogeneity and variation in Mn chemistry in wildland fire ash are highly anticipated because (1) the oxidation of different Mn species is regulated by fire temperature and duration (which are highly variable in the field), as shown in controlled heating of biomass and Mn compounds, (2) fire ash is a mixture of completely and incompletely burned materials that experience different thermal conditions during fires, with Mn being differentially oxidized, and (3) fire behavior is dynamic and variable across ecosystems.

First, wildland fire ash consists of diverse Mn species at multiple valence states and Mn AOS below 4.0, as a result of variable and dynamic fire conditions that produce ash components experiencing different temperatures and durations. Wildland fire ash is physically and chemically heterogeneous, because it is oftentimes a mixture of completely burned inorganics and charred organics that may experience different combinations of temperature, durations, and O_2_ levels.^19^ In addition, fire temperature is spatially variable (ranges from 100 to 1000 °C) and the burning at a site can last from seconds to days.^31,32,54^ Therefore, not all Mn species in biomass are oxidized and some oxidation reactions may not reach equilibrium (some may take hours).^47^ For example, Mn­(II) remains abundant in wildland fire ash (e.g., more Mn­(II) in the char fraction), because the Mn­(II)-embedded ash component may not have reached the temperature and time that are required for Mn oxidation. This is evidenced in the lab heating experiment, which showed that Mn AOS increases with heating durations and burning completeness (Figures 3 and 4).

Second, variable ash Mn chemistry (AOS and Mn speciation) among different ecosystems is likely related to variable fire behaviors, specifically the localized fire temperature and duration of the sampling sites. It is well established that depending on ecosystems and fire weather, different fire types (i.e., crown, surface, and ground fires) and severities may occur, leading to differential consumption of biomass sources, different combustion rates, and fire thermal conditions.^55,56^ The wildland fire ash samples were intentionally selected from highly different ecosystems and originated from wildfire and prescribed fire, presuming with different fire behaviors.^27^ We correlated Mn_XAS_ AOS of wildland fire ash and their total C content (indicative of burning completeness), presuming burning completeness related similarly to fire thermal intensity across ecosystems. However, the two did not correlate (*R* ^2^ = 0.134), suggesting potential variation in fire thermal conditions (fire temperature, duration, and O_2_ level) across ecosystems and their effects on burning completeness and Mn oxidation. For example, high fire temperature and long duration generally lead to a high Mn AOS in fire ash but may not result in the same burning completeness in two different biomass types (e.g., grass and woody biomass). Because fire behavior data is limited (especially localized thermal conditions) for the wildland fires, we cannot further determine the causes of the observed variation in Mn AOS among the field samples. It is worth noting that fire behavior is spatially heterogeneous within individual fires and can vary substantially among different fires. The correlation between Mn AOS, ash burning completeness, and fire thermal intensity (temperature × duration) may not be uniform among ecosystems. Future studies measuring local thermal conditions at ash sampling sites are needed to determine how fire thermal conditions regulate ash Mn chemistry in the field.

### 4.3 Oxidative Reactivity of Fire Ash

Manganese is a redox-active element and exists in diverse mineral phases that are either geogenic or biogenic.^57,58^ The redox reactivity of various Mn oxides has been extensively characterized and depends on factors including (but not limited to) the Mn valence state, mineralogy, and structural incorporation of metals.^59,60^ Our result showed that fire ash can degrade catechol, and the oxidized Mn in ash is responsible for the catechol degradation (Figure 5). In the selected ash samples, Mn exists as primarily Ca_2_Mn_3_O_8_ (AOS = 4.0) or a mixture of Mn species with AOS = 3.3. Although the oxidized Mn is embedded in a complex ash matrix, it remains reactive toward organic structures. We also showed that the oxidative reactivity of fire ash most likely depends on Mn concentration and speciation (i.e., AOS, mineralogy, and physical forms) of Mn in fire ash, with ash with a higher Mn concentration and AOS exhibiting greater reactivity (Figure 5). The concentration of Mn in fire ash is a collective result of the initial Mn content in the fuel biomass and the combustion rate in a fire (which determines the enrichment factor). The degree of Mn oxidation and Mn speciation is primarily controlled by fire thermal conditions, as discussed above. Our spectro-microscopic data showed that Mn is chemically and physically heterogeneous in fire ash (Figure 2B), which could provide a basis for quantitative evaluation of the effects of these properties (e.g., particle size distribution and matrix composition) on the oxidative reactivity of fire ash.

### 4.4 Implications for Postfire Soil Biogeochemical Processes

Fires can alter biogeochemical processes through the burning of aboveground biomass and the surface deposition of fire ash. Understanding the fate and transport of fire ash and its role in postfire biogeochemical processes is an integral part of understanding the response of ecosystems to fire disturbance. Results from this study first reveal a new fire-induced Mn recycling process that is different from that in the absence of fire. In the absence of fires, microbes mediate the decomposition of aboveground biomass in terrestrial ecosystems, particularly in forests,^61^ during which the biomass Mn is gradually oxidized by microbes and involved in important biogeochemical processes in forest floor and topsoils (e.g., litter decomposition).^2,62^ The immediate burning of biomass and oxidation of Mn by fire will change the pathways and forms of Mn returning to soils. Our findings on the chemistry of Mn in fire ash and the effects of fire thermal conditions provide a mechanistic insight into the fire-disturbed Mn cycling in vegetated ecosystems, specifically how the aboveground biomass Mn pool is oxidized.

This study will also shed light on the biogeochemical processes in postfire environments, considering the ecological roles of Mn in vegetated ecosystems. In particular, considering the large amounts of living and dead biomass that can be burned during a fire (e.g., with fuel consumption up to 100s ton·ha^–1^)^55^ and the enrichment and physicochemical forms of Mn in ash as compared to litter, the ash deposited on the ground represents a significant oxidized Mn pool that may change the paradigm of organic decomposition on the forest floor and topsoil. For example, the oxidized Mn in the organic layer (generated slowly by microbes) represents a relatively small Mn pool in the soil profile, yet it plays a critical role in litter and soil organic matter decomposition.^62,63^ The immediate deposition of a large amount of oxidized Mn, whose phases and structures are different from those of biogenic Mn oxides, is expected to change the content and chemistry of Mn along the soil profile, potentially affecting the relevant biogeochemical processes mediated by Mn. In this regard, our findings of Mn chemistry in fire ash and its oxidative reactivity will provide fundamental knowledge for exploring soil biogeochemical processes, such as organic matter decomposition, in postfire environments.

## Supplementary Material



## Notes

Details of biomass and ash collection and preparation; details of chemical analysis of total and extractable Mn; general information on the fire ash samples from prescribed fire and wildfire; Mn K-edge XANES spectra of references and ash samples; and results of the Combo fitting of the first derivative XANES data and EXAFS shell-by-shell fitting (PDF)
