# Supplementary material for: Manganese Oxidation during Vegetation Burning
Source: Environ Sci Technol. 2026 Jun 1;60(23):16641–50. doi: 10.1021/acs.est.6c05048 (PMC13276906; doi:10.1021/acs.est.6c05048)
Supplement: Supplementary file 1 [file es6c05048_si_001.pdf]

Supplementary information

Manganese oxidation during vegetation burning

Shyrill Mae F. Mariano[1], Lingqun Zeng[1], Rixiang Huang[1*], Carmen Sánchez-García[2,3], Cristina Santin[3,4], Jonay Neris[3,5], Peng Yang[6], Lu Ma[6], and Andrew Kiss[6]

1. Department of Environmental and Sustainable Engineering, University at Albany 1400 Washington Ave, Albany, New York, 12222, USA
2. European Commission, Joint Research Centre (JRC), Ispra, 21027, Italy
3. Centre for Wildfire Research, Swansea University, Swansea, SA2 8PP, UK
4. Research Institute of Biodiversity (IMIB; CSIC-UnOvi-PA), University Campus of Mieres, Mieres, 33600, Spain
5. Universidad de La Laguna, Tenerife, 38206, Spain
6. National Synchrotron Light Source II, Brookhaven National Laboratory, Upton, New York, 11973, USA

*Corresponding Author:
Email: rhuang6@albany.edu
Phone: 518-437-4977

Numbers of pages: 15

Numbers of text: 3

Numbers of Tables: 4

Numbers of Figure: 5

Text S1. Sample preparation.

The biomass feedstock used for laboratory burning are compartments (leaf, cone, and stem) of black spruce and white pine needle. The biomass was litter collected from the Albany Pine Bush Preserve (APBP) at Albany, NY. Specifically, litter that was newly fell on the ground was collected for the laboratory burning and the biomass was pooled from at least three distanced spots. The biomass was dried in an oven at 80 °C for at least 3 days until no further weight change. The dried biomass was pulverized by a blender for subsequent use. The laboratory burning was done by burning the uniform biomass in a furnace in air at 450 to 700 °C for varying durations (5 min to 5 hours).

Prescribed fire ash samples were collected from the Albany Pine Bush Preserve (APBP, Albany, NY). One prescribed fire was conducted at a grass-dominated site in 12/14/2021 (the left figure below), another one was conducted at a wood-dominated site (mix of bitch pine and oak) in 11/20/2023 (the right figure below), and the third was a bitch pine dominated site (fire conducted in June 2022). The samples were collected immediately after the prescribed fires (within a few hours and without precipitation) from ground surface using a scoop. The samples were pooled randomly from multiple sites (at least five) across the burning area.

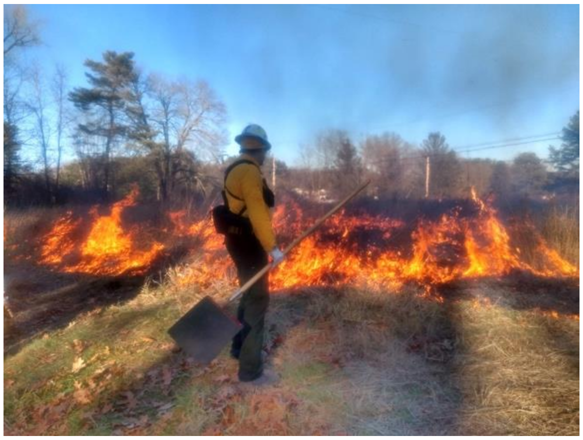

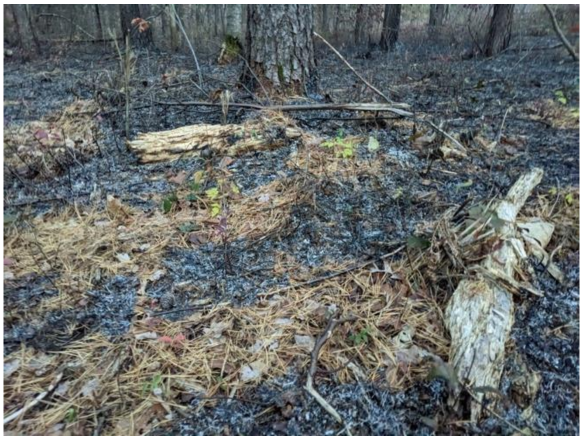

Text S2. Analysis of total and available Mn.

For the analysis of total Mn ($[Mn]_{total}$), ash and biomass samples ranging between 20 to 50 mg were digested in aqua regia overnight. The digestates were diluted to 5 mL with deionized water and filtered with a 0.45 μM syringe filter, then an aliquot of 0.5 to 2 mL was added with a 0.1 mL of 100 ppm internal standard, before bringing to a final volume of 10 mL with 2% nitric acid. Bioavailable Mn ($[Mn]_{Pyro}$%) was quantified through a modified pyrophosphate extraction, which extracts both Mn^{2+} and Mn^{3+} in the forms of soluble and organic-complexed Mn [1]. A 2 mg sample was mixed with 1.5 mL 0.1 M K-pyrophosphate at pH 10 and was agitated in the dark for 8 h at 150 RPM. The samples were centrifuged at 17,000 G for 15 minutes and were filtered through a 0.45 μM syringe filter. An aliquot of 0.5 mL was diluted to 10 mL with 2% HNO_3 after addition of 0.25 mL internal standard. The samples were then analyzed using an ICP-OES. Duplicate experiments were done for the analysis of total Mn and bioavailable Mn, and the mean was reported (Table S1).

Text S3. Data collection for X-ray absorption spectroscopic analysis.

Data collection. SSRL Beamline 11-2 used a "double double" Si (220) LN_2-cooled monochromator and a 100-pixel monolithic solid-state Ge detector array, whereas NSLS-II Beamline 6-BM operated with a Si(111) monochromator and a 7-element Vortex detector. Metallic Mn placed between the rear ionization chambers was used for energy calibration and was measured between every 5 samples to check energy stability. The first inflection point of the K-edge energy was set to 6539.0 eV.

For the analysis in SSRL, fine powders of the biomass and ash samples were uniformly packed on metal sample holders and sealed on both sides with a Kapton tape. The multi-sample holder loaded with samples was placed at a 45° angle with the incident ray and analyzed with the fluorescence mode under vacuum. For the analysis in NSLS-II, samples were thinly spread on a Kapton tape and was folded for ~3 times to an appropriate thickness, then mounted on a rotatory sample holder. The XAS spectra from SSRL were collected from 6310.0 to 6937.2 eV, with the following steps: 10 eV for 6310.0 eV to 6520.0 eV, 0.35 eV for 6520.0 eV to 6570.0 eV, and 0.1 k for 6570.0 eV. The XANES data from NSLS-II were collected from 6339 to 7084 eV, with the following steps: 10 eV for 6339.0 eV to 6509.0 eV, 2 eV for 6509.0 to 6529.0 eV, 0.3 eV for 6529.0 to 6564.0 eV, and 0.2 k beyond 6564.0 eV. Extended X-ray absorption fine structure (EXAFS) was collected for selected samples with relatively high Mn contents, using 0.05 k for energy beyond 6564.0 eV. Two to four scans were collected for each sample.

XANES analysis. Collected spectra were aligned, averaged, and calibrated using SixPack and ATHENA softwares.[2,3] Principal component analysis and target transformation were performed to select the appropriate monovalent Mn^{4+}, Mn^{3+}, and Mn^{2+} reference standards, before linear combination fitting (LCF) of the first derivatives from 6530.0 to 6650.0 eV, following the Combo method using the first derivative to estimate Mn average oxidation state.[4] Reference standards were set to return non-negative loadings, and those with less than 5% contribution were sequentially removed. Reported Mn AOS was derived from the contributions of the remaining reference standards which weights were forced to 1. The fitting with the smallest normalized sum-squared residuals (NSS) value was deemed the best fit.[4]

EXAFS analysis. EXAFS data analysis was done in ARTEMIS software[3] for suitable samples to identify specific Mn species. Least squares fitting was conducted over a k range of 3.0 to 11.0Å, where coordination number (CN), energy shift (ΔE_0), or amplitude factor (S_0^2) were kept constant while iterating the mean half path length (R) and Debye-Waller factor (σ^2). Amplitude was determined by multiplying CN with the S_0^2. For samples scanned with a reference metal Mn foil, S_0^2 was assigned by fitting the first Mn shell and obtaining the value that gave the best fit. S_0^2 was then kept constant while fitting ΔE_0. Coordination numbers were fixed based on literature values of the suspected Mn reference, and energy shift was adjusted accordingly while maintaining the Debye-Waller factor below 0.01. An R-factor below 0.05 was considered a reasonable fit. An F-test was additionally conducted for selected samples to assess whether a change in the model by adding another shell will improve the fit [5].

Table S1. General information of the tested fire ash samples from prescribed fire and wildfire.

Sample label	Ecosystem/Dominant Vegetation	Location	Fire type	Fire severity	Mn_{XAS} AOS	Mn_{LBB} AOS	$[Mn]_{Total}$ (mg/kg) [6]	$[Mn]_{pyro}$ (%)
Eucalypt	Temperate eucalypt forest (dry) / Dry eucalypt forest with dense shrubby understory	Australia	Experimental	High	2.9	3.5	400	55.3
Boreal	Boreal forest/ Mature jack pine	Canada	Experimental	High	2.5	2.5	450	62.5
Savanna	Sub-tropical savanna/ Knobthorn-Marula	South Africa	Experimental	Low	2.6	NA	300	32.9
Heath-Sp	Temperate heathland/ Heathland	Spain	Wildfire	High	2.9	3.4	1100	29.7
Heath-UK	Temperate heathland/ Upland moorland	UK	Wildfire	High	2.7	2.5	500	41.3
Temperate	Temperate conifer forest/ Mixed conifer forest	USA	Wildfire	Low	3.1	3.3	1900	17.3
APBP-Grass	Pine Barrens/Grass	Albany, NY	Prescribed	Low	2.8	2.4	1700	38.7
APBP-W1	Pine Barrens/Wood	Albany, NY	Prescribed	Low	3.3	3.5	5400	19.3
APBP-W2	Pine Barrens/Wood	Albany, NY	Prescribed	Low	3.2	4.0	4500	30.7

Note: Fire severity was determined based on the characteristics of the sampling area: ash color and degree of vegetation combustion.[7]

Table S2. Chemical formula, nominal Mn average oxidation state (AOS), and literature sources of Mn reference compounds for species estimation.

	Mn reference compound	Chemical formula	Structure	Nominal AOS	Reference
1	Na-birnessite (synthetic)	MnO_2	triclinic	3.92	[8]
2	K-birnessite	$K^{+}_{0.296}(Mn^{4+}_{0.926}□_{0.074})O_2 \cdot 0.40H_2O$		4.00	[4]
3	Todorokite	$(Mn^{2+},Ca,Na,K)(Mn^{4+},Mn^{2+},Mg)_6O_{12} \cdot 3H2O$	3x3 tunnel tectomanganate	3.67	[9]
4	$Ca_2Mn_3O_8$	$2/3\ Ca_2Mn_3O_8 + 1/3\ CaMnO_3$		4.00	[4]
5	δ-MnO2	$(Mn^{4+},Fe^{3+},Ca,Na)_{\Sigma 1.00}(O,OH)_2 \cdot nH2O$	phyllomanganate	4.00	[9]
6	Bixbyite	Mn_2O_3	cubic	3.00	[9]
7	Feitknechtite	β-MnOOH	octahedral	3.00	[9, 10]
8	Hausmannite	Mn_3O_4	tetragonal	2.67	[9]
9	Triplite	$(Mn^{2+},Fe^{2+},Ca,Mg)_2(PO_4)(F,OH)$	phosphate	2.00	[9]
10	Mn(II) adsorobed on illite	$K_{0.65}Al_{2.0}(Al_{0.65}Si_{3.35}O_{10})(OH)_2 \cdots Mnx^{2+}$	phyllosilicate	2.00	[9]
11	Mn(II) adsorbed on peat		organic sediment	2.00	[9]
12	Mn(II) oxalate dihydrate	$MnC_2O_4 \cdot 2H2O$	monoclinic	2.00	[9]
13	Mn(II) acetate tetrahydrate	$(CH_3COO)_2Mn \cdot 4H_2O$	-	2.00	[9]

* □ denotes vacancy.

Table S3. Results from the Combo fit of the XANES first derivative of ash samples between 6530 – 6650 eV interval.

Sample	Mn4+ Reference Standards					Mn3+ Reference Standards					Mn2+ Reference Standards					TOTAL	AOS	NSS $x10^{-2}$
	1	2	3	4	*SUM*	1	2	3	4	*SUM*	1	2	3	4	*SUM*			
APBP-Grass			0.116	0.089	*0.205*	0.400				*0.400*			0.394		*0.394*	0.999	**2.81**	0.78
APBP-W1				0.372	*0.372*	0.537				*0.537*	0.091				*0.091*	1	**3.28**	0.78
APBP-W2		0.156		0.148	*0.304*	0.514				*0.514*			0.182		*0.182*	1	**3.21**	0.41
Temperate	0.421	0.245			*0.666*	0.2				*0.2*			0.134		*0.134*	1	**3.03**	1.25
Boreal					*0*	0.203			0.268	*0.471*			0.428	0.101	*0.529*	1	**2.6**	2.10
Heath-Sp	0.214				*0.214*	0.457				*0.457*			0.329		*0.329*	1	**2.94**	0.59
Heath-UK	0.109				*0.109*	0.273			0.221	*0.494*			0.398		*0.398*	1.001	**2.78**	2.40
Savanna					*0*		0.327	0.308		*0.635*		0.084	0.28		*0.364*	0.999	**2.72**	2.24
Eucalypt	0.175				*0.175*	0.302			0.215	*0.517*			0.308		*0.308*	1	**2.76**	2.24
Wood >125 µm			0.252		*0.252*			0.297		*0.297*		0.293	0.158		*0.451*	1	**2.80**	1.83
Wood <125 µm			0.217	0.091	*0.308*	0.568				*0.568*		0.125			*0.125*	1.001	**3.19**	2.11
Grass >125 µm		0.146			*0.146*	0.470		0.309		*0.779*				0.075	*0.075*	1	**3.07**	5.23
Grass <125 µm	0.336				*0.336*	0.390		0.210		*0.600*	0.064				*0.064*	1	**3.27**	0.79
WPN 5m 550					*0*	0.103			0.594	*0.697*		0.302			*0.302*	0.999	**2.70**	1.40
WPN 20m 550	0.136				*0.136*	0.523				*0.523*	0.137			0.204	*0.314*	1	**2.80**	1.05
WPN 30m 550	0.367		0.168		*0.535*	0.300				*0.300*			0.165		*0.165*	1	**3.37**	1.02
WPN 5h 550	0.841		0.159		*1.000*					*0*					*0*	1	**4.00**	1.37
WPN 5m 700	0.484		0.190		*0.674*	0.259				*0.259*		0.066			*0.066*	1	**3.61**	0.85
WPN 20m 700	0.498		0.217		*0.715*	0.231				*0.231*				0.084	*0.084*	1	**3.60**	0.74
WPN 5h 450	0.802		0.198		*1*					*0*					*0*	1	**4.00**	1.39
WPN 5h 600	0.330		0.435		*0.765*	0.235				*0.235*					*0*	1	**3.77**	1.12
BS Leaf biomass					*0*				0.282	*0.282*			0.718		*0.718*	1	**2.29**	7.91
BS Leaf 5m 550					*0*	0.235			0.363	*0.598*		0.147	0.255		*0.402*	1	**2.60**	1.21
BS Leaf 20m 550	0.442				*0.442*	0.312				*0.312*			0.155	0.091	*0.246*	1	**3.20**	0.62
BS Leaf 30m 550	0.512		0.082		*0.594*	0.247				*0.247*				0.160	*0.160*	1.001	**3.44**	0.79

BS Leaf 5h 550	0.79	0.21			*1*					*0*					*0*	1	**3.77**	0.61
BS Stem biomass					*0*				0.295	*0.295*			0.606	0.099	*0.705*	1	**2.33**	6.08
BS Stem 20m 550	0.279				*0.279*	0.300			0.195	*0.495*		0.225			*0.225*	1	**3.05**	1.09
BS Stem 5h 550	0.697	0.303			*1*					*0*					*0*	1	**4**	1.69
BS Cone biomass					*0*				0.738	*0.738*			0.262		*0.262*	1	**2.76**	5.66
BS Cone 5h 550	0.774		0.127		*0.901*					*0*			0.099		*0.099*	1	**3.74**	0.96
Mn(II) Ace 5h 450	0.261		0.224		*0.485*	0.298				*0.298*		0.216			*0.216*	0.999	**3.27**	1.12
Mn(II) Ace 5h 600		0.202		0.098	*0.300*	0.250		0.451		*0.700*					*0*	1.001	**3.30**	1.23

Note: Different sample categories were color coded. Orange – wildland fire ash; Green – lab burned ash of white pine needle; Blue - lab burned ash of black spruce biomass. Sample name includes the heating duration and temperature.

Monovalent Mn standards: Mn^{4+}: 1) KBi (synthetic birnessite), 2) Ramsdellite, 3) Pyrolusite, 4) Ca2Mn3O8; Mn^{3+}: 1) Feitknechtite, 2) Groutite, 3) Mn2O3, 4) MnPO4; Mn^{2+}: 1) Pyroxmangite, 2) Hureaulite, 3) Mn(II) acetate/oxalate, 4) Tephroite.

NSS (normalized sum-squared residual) = $\sum residual^2 / \sum data$

Table S4. Results from the k^2-weighted EXAFS shell-by-shell fitting of selected samples between 3-11 Å.

Sample	ΔE_0	Amplitude factor	Atom (CN)	R	σ^2	*R*-factor
APBP-W1	-9.3 (1.5)	0.65	O1: 4	1.84	0.009 (0.001)	0.020
			O2: 2	2.36	0.004 (0.004)	
			Mn1: 1	2.70	0.009 (0.006)	
			Mn2: 1	2.92	0.005 (0.005)	
			O3: 4	3.40	0.002 (0.003)	
			O4: 4	4.12	0.002 (0.004)	
APBP-W1 14d	-3.8 (1.2)	0.58	O1: 2	1.92	0.005 (0.002)	0.017
			O2: 4	2.15	0.007 (0.001)	
			O3: 4	3.38	0.008 (0.002)	
			Mn1: 2	3.77	0.004 (0.002)	
BS Leaf 5m 550	-4.7 (1.5)	0.58	O1: 3	1.94	0.006 (0.001)	0.016
			O2: 3	2.13	0.001 (0.003)	
			Mn1: 1	2.98	0.004 (0.002)	
BS Leaf 20m 550	0.6 (1.3)	0.58	O1: 4	1.92	0.006 (0.001)	0.016
			O2: 2	2.19	0.001 (0.003)	
			Mn1: 2	2.88	0.004 (0.002)	
			Mn2:4	3.07	0.009 (0.002)	
BS Leaf 30m 550	-1.6 (1.3)	0.58	O1: 5	1.92	0.006 (0.001)	0.015
			O2: 1	2.23	0.001 (0.003)	
			Mn1: 2	2.88	0.004 (0.002)	
			Mn2:4	3.06	0.009 (0.002)	
BS Leaf 5h 550	-0.4 (1.0)	0.40	O1: 6	1.90	0.003 (0.001)	0.010
			Mn: 4	2.92	0.005 (0.001)	
			Ca: 4	3.18	0.004 (0.003)	
			O2: 4	3.60	0.008 (0.005)	
BS Stem 20m 550	-2.1 (0.9)	0.58	O1: 4	1.94	0.009 (0.001)	0.007
			O2: 2	2.18	0.007 (0.001)	
			Mn1: 1	2.86	0.007 (0.002)	

			Mn2:2	3.04	0.005 (0.001)	
BS Stem 5h 550	-5.4 (1.1)	0.50	O1: 6	1.91	0.004 (0.001)	0.012
			Mn: 4	2.96	0.007 (0.001)	
			Ca: 4	3.23	0.007 (0.002)	
			O2: 4	3.75	0.005 (0.005)	
WPN 5m 550	1.1 (1.6)	0.50	O1: 4	2.07	0.007 (0.001)	0.016
			O2: 2	2.25	0.007 (0.003)	
			Mn1: 1	2.99	0.006 (0.004)	
			Mn2: 2	3.16	0.004 (0.004)	
			O3: 1	3.89	0.006 (0.014)	
WPN 20m 550	-2.7 (1.1)	0.50	O1: 4	1.94	0.006 (0.001)	0.025
			O2: 2	2.18	0.0002 (0.001)	
			Mn1: 1	2.91	0.009 (0.006)	
			Mn2:2	3.08	0.006 (0.002)	
			O3: 2	3.77	0.002 (0.003)	
WPN 30m 550	-0.8 (1.1)	0.58	O1: 5	1.91	0.008 (0.001)	0.019
			O2: 1	2.21	0.001 (0.002)	
			Mn1: 2	2.90	0.008 (0.002)	
			Mn2: 4	3.08	0.009 (0.002)	
			O3: 2	3.69	0.008 (0.008)	
WPN 5h 550	-6.2 (1.3)	0.50	O1: 6	1.91	0.004 (0.001)	0.011
			Mn: 4	2.95	0.008 (0.002)	
			O2: 4	3.06	0.006 (0.002)	
			Ca: 4	3.24	0.005 (0.006)	
WPN 5m 700	-5.1 (1.1)	0.58	O1: 5	1.91	0.006 (0.001)	0.012
			O2: 1	2.28	0.002 (0.003)	
			Mn1: 2	2.92	0.004 (0.001)	
			Mn2: 4	3.10	0.008 (0.001)	
			O3: 4	3.72	0.005 (0.003)	
WPN 20m 700	-3.5 (1.0)	0.58	O1: 5	1.91	0.007 (0.002)	0.008
			O2: 1	2.31	0.001 (0.002)	
			Mn1: 2	2.95	0.005 (0.006)	
			Mn2:4	3.10	0.001 (0.003)	

			O3: 4	3.80	0.005 (0.007)	
WPN 5h 450	-9.3 (1.4)	0.50	O1: 6	1.88	0.003 (0.001)	0.010
			Mn: 4	2.95	0.008 (0.002)	
			Ca: 4	3.43	0.005 (0.003)	
			O2: 4	3.57	0.005 (0.005)	
WPN 5h 600	-6.9 (1.7)	0.50	O1: 6	1.90	0.003 (0.001)	0.017
			Mn: 4	2.94	0.007 (0.002)	
			Ca: 4	3.20	0.003 (0.002)	
			O2: 4	3.76	0.002 (0.005)	
WPN500 21d	-3.7 (1.4)	0.58	O1: 2	1.93	0.007 (0.002)	0.023
			O2: 4	2.17	0.005 (0.001)	
			Mn1: 1	2.93	0.006 (0.004)	
			Mn2: 1	3.11	0.008 (0.006)	

Uncertainties are denoted within a parenthesis; otherwise, the parameter was constrained during the fit.

ΔE_0: Energy shift parameter; Amplitude factor: Parameter multiplied to CN to obtain S_0^2 ; CN_n: Coordination number of nth shell; σ^2: Debye-Waller parameter; R: Mean half path length. Samples listed are as follows: BA – black spruce ash, WPN – white pine needle ash.

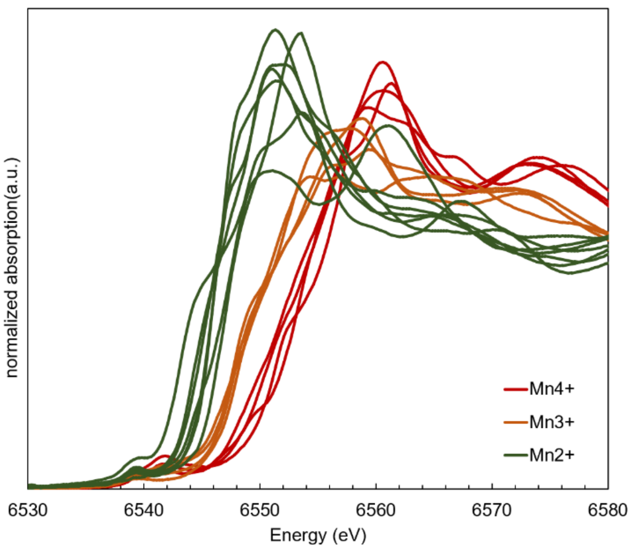

Figure S1. Mn XANES spectra of 15 reference monovalent Mn^{2+}, Mn^{3+}, and Mn^{4+} species used for Combo fitting.[11]

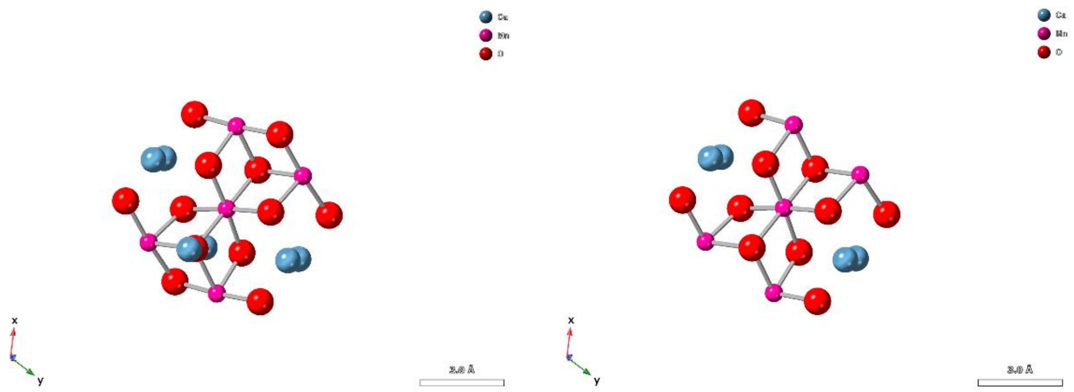

Figure S2. Crystallographic structure of $Ca_2Mn_3O_8$ reference (left) and $Ca_2Mn_3O_8$-like Mn oxide identified in complete burning ash (right).

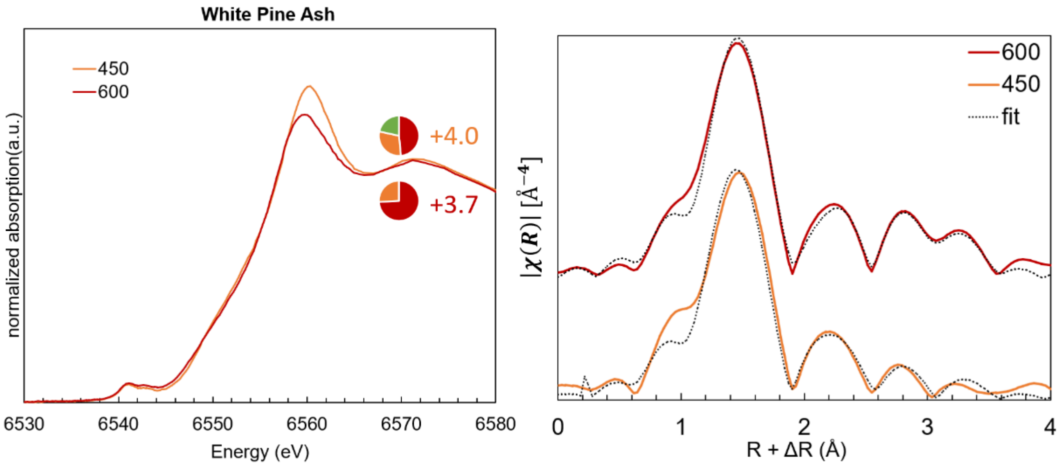

Figure S3. Mn K-edge XAS of WPN ash at 450 °C and 600 °C for 5 h (right: EXAFS fitting of 450 °C and 600 °C with $Ca_2Mn_3O_8$).

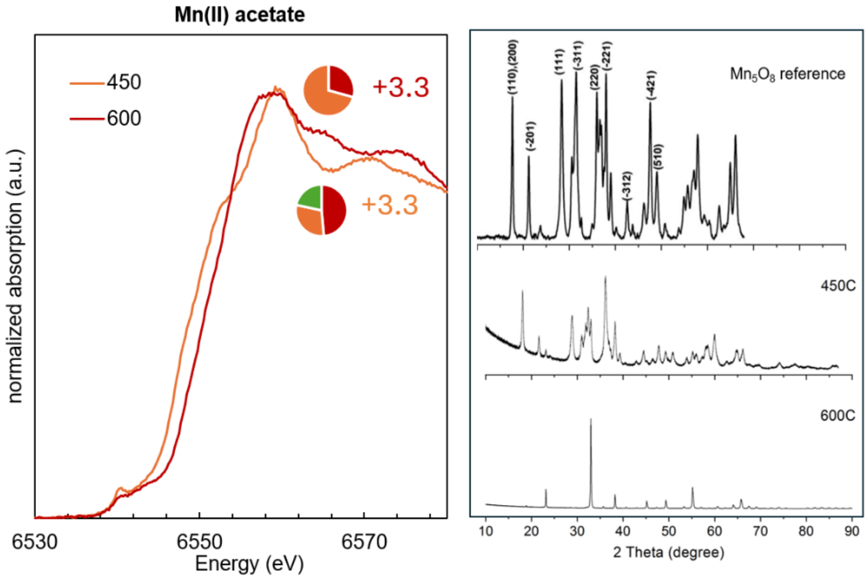

Figure S4. (Left) Mn K-edge XAS of Mn-Acetate at 450 °C and 600 °C for 5 h. (Right) XRD profile of Mn_5O_8 from published literature [12] and Mn(II) acetate heated at 450, and 600 °C.

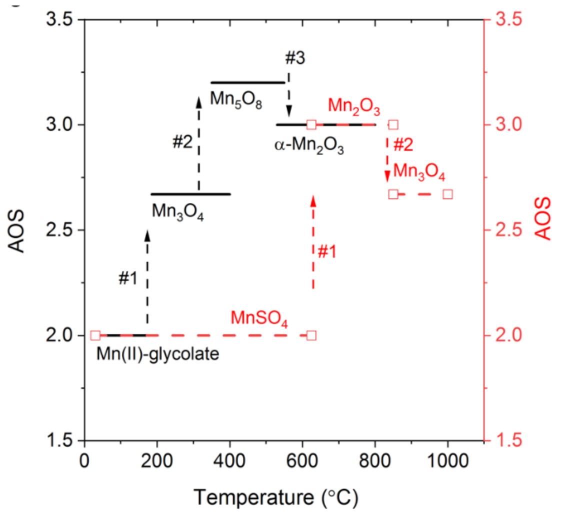

Figure S5. Transformation of Mn(II)-glycolate and Mn sulfate flowing ramp heating in the presence of O_2. Data were based on results of Augustine et al. and Warner et al.[13, 14]

References

1. Keiluweit, M.; Nico, P.; Harmon, M. E.; Mao, J.; Pett-Ridge, J.; Kleber, M., Long-term litter decomposition controlled by manganese redox cycling. *Proc Natl Acad Sci U S A* **2015,** *112*, (38), E5253-60.
2. Webb, S. M.; Webb, S. M., SIXpack: a graphical user interface for XAS analysis using IFEFFIT. *Physica Scripta* **2005-01-01,** *2005*, (T115).
3. Ravel, B.; Newville, M., ATHENA, ARTEMIS, HEPHAESTUS: data analysis for X-ray absorption spectroscopy using IFEFFIT. *Journal of synchrotron radiation* **2005,** *12*, (4), 537-541.
4. Manceau, A.; Marcus, M. A.; Grangeon, S., Determination of Mn valence states in mixed-valent manganates by XANES spectroscopy. *American Mineralogist* **2012,** *97*, (5-6), 816-827.
5. Downward, L.; Booth, C.; Lukens, W.; Bridges, F. In *A variation of the F-test for determining statistical relevance of particular parameters in EXAFS Fits*, AIP Conference Proceedings, 2007; American Institute of Physics: 2007; pp 129-131.
6. Zeng, L.; Mariano, S. F.; Huang, R.; Sánchez-García, C.; Santin, C.; Neris, J.; Kumar, K.; Glenn, C. K.; El Hajj, O.; Anosike, A.; O'Brien, J.; Saleh, R. A., Speciation and Aqueous Dissolution of Macronutrients in Fire Ash: Variation across Ecosystems and the Effects on Nutrient Cycling. *Environ Sci Technol* **2025,** *59*, (1), 454-466.
7. Sánchez-García, C.; Santín, C.; Neris, J.; Sigmund, G.; Otero, X. L.; Manley, J.; González-Rodríguez, G.; Belcher, C. M.; Cerdà, A.; Marcotte, A. L.; Murphy, S. F.; Rhoades, C. C.; Sheridan, G.; Strydom, T.; Robichaud, P. R.; Doerr, S. H., Chemical characteristics of wildfire ash across the globe and their environmental and socio-economic implications. *Environment International* **2023,** *178*, 108065.
8. Villalobos, M.; Toner, B.; Bargar, J.; Sposito, G., Characterization of the manganese oxide produced by pseudomonas putida strain MnB1. *Geochimica et Cosmochimica Acta* **2003,** *67*, (14), 2649-2662.
9. Zahoransky, T.; Wegorzewski, A. V.; Huong, W.; Mikutta, C., X-ray absorption spectroscopy study of Mn reference compounds for Mn speciation in terrestrial surface environments. *American Mineralogist: Journal of Earth and Planetary Materials* **2023,** *108*, (5), 847-864.
10. Post, J. E.; Heaney, P. J.; Ilton, E. S.; Elzinga, E. J., The crystal structure of feitknechtite (β-MnOOH) and a new MnOOH polymorph. *American Mineralogist* **2023,** *108*, (11), 2131-2141.
11. Manceau, A.; Marcus, M. A.; Grangeon, S.; Manceau, A.; Marcus, M. A.; Grangeon, S., Determination of Mn valence states in mixed-valent manganates by XANES spectroscopy. *American Mineralogist* **2012-05-01,** *97*, (5-6).
12. Zhang, P.; Liu, J.; Page, K.; Navrotsky, A., Calorimetric study of the thermodynamic properties of Mn₅O₈. *Journal of the American Ceramic Society* **2019,** *102*, (3), 1394-1401.
13. Warner, T. E.; Bancells, M. M.; Lund, P. B.; Lund, F. W.; Ravnsbæk, D. B., On the thermal stability of manganese(II) sulfate and its reaction with zeolite A to form the sodalite Na6Mn2[Al6Si6O24](SO4)2. *J Solid State Chem* **2019,** *277*, 434-440.
14. Augustin, M.; Fenske, D.; Bardenhagen, I.; Westphal, A.; Knipper, M.; Plaggenborg, T.; Kolny-Olesiak, J.; Parisi, J., Manganese oxide phases and morphologies: A study on calcination temperature and atmospheric dependence. *Beilstein J Nanotech* **2015,** *6*, 47-59.
